# Variability of microcirculatory measurements in healthy volunteers

**DOI:** 10.1038/s41598-022-22947-x

**Published:** 2022-11-18

**Authors:** M. E. Bol, B. E. K. Broddin, T. Delhaas, J. E. M. Sels, M. C. G. van de Poll

**Affiliations:** 1 Department of Intensive Care Medicine, Maastricht University Medical Center, MUMC+), Maastricht, The Netherlands; 2 School of Nutrition and Translational Research in Metabolism (NUTRIM), Maastricht University, Maastricht, The Netherlands; 3 Department of Biomedical Engineering, Cardiovascular Research Institute Maastricht (CARIM), Maastricht University, Maastricht, The Netherlands; 4 Cardiovascular Research Institute Maastricht (CARIM), Maastricht University, Maastricht, The Netherlands; 5 Department of Cardiology, Maastricht University Medical Center, MUMC+), Maastricht, The Netherlands; 6 Department of Surgery, Maastricht University Medical Center, MUMC+), Maastricht, The Netherlands

**Keywords:** Medical research, Cardiovascular diseases, Blood flow

## Abstract

Reliable assessment of the microcirculation is important to investigate microcirculatory properties in various disease states. The GlycoCheck system automatically analyzes sublingual sidestream dark field images to determine the perfused boundary region (PBR; a measure of glycocalyx thickness), red blood cell filling percentage, and microvascular vessel density. Although GlycoCheck has been used to study the microcirculation in patients, little is known about the reproducibility of measurements in healthy volunteers. We assessed intra- and interobserver agreement by having two experienced observers perform three consecutive microcirculation measurements with the GlycoCheck system in 49 healthy volunteers. Intraobserver agreement of single measurements were poor (intraclass correlation coefficients (ICCs) < 0.4) for PBR, red blood cell filling percentage and microvascular vessel density. ICCs increased to values > 0.6 (indicating good reproducibility) for all parameters when performing and averaging three consecutive measurements. No systematic differences were observed between observers for any parameter. Interobserver variability was fair for PBR (ICC = 0.53) and red blood cell filling percentage (ICC = 0.58) and poor for perfused vessel density (ICC = 0.20). In conclusion, GlycoCheck software can be used with acceptable reliability and reproducibility for microcirculation measurements on a population level when averaging three consecutive measurements. Repeated measurements are preferably performed by the same observer.

## Introduction

Actual exchange of oxygen, nutrients and waste products takes place in the microcirculation, consisting of blood vessels of < 250 μm in diameter (microvessels)^1^. The glycocalyx lines the luminal side of the microvessels and forms a semi-permeable barrier between blood and endothelium^2^. It plays an important role in the regulation of coagulation factors, prevention of leakage of plasma components, activation/inhibition of platelets and adhesion blocking of leukocytes^3,4^. The role of the microcirculation and specifically of the glycocalyx in the pathophysiology of different disease states is increasingly acknowledged^5,6^.

Reliable assessment of the microcirculation and its pathology is important to investigate its properties in various disease states^5,6^. Eventually, this may lead to the implementation of microcirculatory monitoring as an adjunct to guide therapy. Microcirculation can be assessed non-invasively and in vivo using one of the following video microscopy techniques: orthogonal polarized spectral imaging, side-stream dark field (SDF) imaging, and incident dark field (IDF) imaging^7^. The application of these video microscopes is currently limited to scientific research due to several factors. First of all, knowledge on reference values in different populations is limited. Secondly, validated microcirculatory measurement targets that can influence clinical outcome are lacking^6,8,9^. To develop such targets and move towards the implementation of microcirculatory-guided therapy in clinical practice, a validated, readily applicable and reliable assessment tool is imperative. Most camera systems, however, rely on an off-line, manual analysis of the captured videos to determine microcirculatory variables. This is time consuming and requires thoroughly trained staff.

GlycoCheck (Microvascular Health Solutions Inc., Salt Lake City, UT, USA) is a commercially available software package that automates assessment of the microcirculation based on SDF. It selects and analyses SDF images that are of sufficient quality (adequate focus, adequate contrast and limited movement) and determines glycocalyx thickness, microvascular vessel density and red blood cell filling percentage^10^. Unfortunately, inter- and intraobserver agreement of this technique when used to assess the microcirculation in pathological conditions, is poor^11–13^. Averaging three consecutive measurements was shown to achieve intraobserver variability that can be classified as “excellent” in pathological conditions such as critical illness and smoking^11,12^. Whether this is due to the biological variability caused by pathological conditions or due to the inherent inaccuracy of GlycoCheck system is yet to be determined. The aim of the present study was to assess the reproducibility of GlycoCheck measurements in young, healthy volunteers without any pathological condition that affects microcirculation. To this effect, we evaluated both intra- and interobserver agreement.

## Methods

We included non-smoking, healthy volunteers between 18 and 40 years of age. Exclusion criteria were diabetes mellitus type 1 or 2, nicotine-use and chronic illness for which chronic medication is used. Additionally, volunteers were excluded in case of oral bleeding, oral wounds and oral infections as these reduce the measurement quality. We recorded age, sex, height and weight of the volunteers.

### Measurements

Measurements were performed using an SDF camera (CapiScope HVCS, KK Technology, Honiton, UK) fitted with GlycoCheck software (Microvascular Health Solutions Inc., Salt Lake City, UT, USA). Two researchers (MEB and BB), both experienced in performing sublingual measurements with this tool, performed three consecutive measurements for each subject. The measurement order of the two researchers was randomly determined. Measurements were taken with subjects in supine position with the researcher standing behind the headboard. Volunteers were asked to swallow any saliva prior to measuring after which the camera was manually placed and held still in the sublingual region. To limit movement of the camera, the researchers could rest their wrist on the lower jaw of the volunteers. Care was taken to limit pressure artifacts by ensuring erythrocytes could be seen traveling through the blood vessels during the measurements. Images were recorded only in the absence of air bubbles, excessive amounts of saliva, excessive loops of the vessels and excessive amounts of large venules. All measurements in an individual volunteer were performed within 30 min.

#### GlycoCheck parameters

The GlycoCheck software records movies of 1 s that consist of 23 frames. Recording is initiated automatically when the software deems the images of sufficient quality, meaning that the intensity and focus are sufficient for calculations and that the camera is held sufficiently still. Vessels are automatically detected and measurement points are defined at 10 µm intervals. GlycoCheck limits its calculations to vessels with a width between 5 and 25 µm. A measurement is complete when 3000 measurement points have been acquired.

##### Perfused boundary region

The inner layer of the glycocalyx is penetrable to red blood cells and, hence, also called the perfused boundary region (PBR)^10^. An intact glycocalyx is thicker and less penetrable to red blood cells, leading to a thinner PBR, compared to a damaged glycocalyx. The PBR is thus an inverse measure of the glycocalyx thickness. GlycoCheck software calculates the PBR from the intensity profile at every measurement point^10^. A more gradual increase of the intensity profile means a thicker PBR – indicating a thinner glycocalyx.

##### Red blood cell filling percentage

The red blood cell filling percentage, a measure of microvascular perfusion, is defined as the median percentage of time that red blood cells are present at each measurement point^10^.

##### Microvascular vessel density

GlycoCheck calculates microvascular vessel density from the amount of measurement points—as each measurement point represents 10 µm of microvessel length^10^. Cumulative microvessel length in µm was thus equal to the amount of measurement points multiplied by 10. Microvascular vessel density in µm/mm^2^ was calculated by dividing the cumulative microvessel length by the total recorded area in mm^2^.

### Statistics

Data are presented as median [25–75th percentile] or as mean ± standard deviation (SD) as appropriate. Normality was assessed using the Kolmogorov–Smirnov test and the Shapiro Wilk tests for normality. Data analysis was performed with SPSS (version 27; IBM, Armonk, NY, USA). P-values < 0.05 were considered to be statistically significant. Volunteers were excluded from the analysis in case of a missing measurement.

Intraobserver agreement for single measurements was assessed with Intraclass Correlation Coefficients (ICCs) by means of a two-way random model with absolute agreement (type ICC(2,1) according to the Shrout and Fleiss convention^14^) and reported as ICC (95% confidence interval; CI) for each of the two observers. For the mean of three measurements ICCs were assessed by means of a two-way random model with absolute agreement for the mean of multiple measurements (type ICC(2, k); where k = 3). ICCs were deemed as poor (< 0.40), fair (0.40–0.60), good (0.60–0.75) or excellent (> 0.75) according to the guidelines written by Cicchetti^15^.

Sample size calculation was performed such that an ICC of 0.6 or higher (indicating good reproducibility) would be detected with a 95% confidence interval width of at most 0.3. Based on the method described by Lew et al., we calculated a sample size of 45 volunteers^16^.

The average values of the 3 measurements taken by each observer were used for interobserver agreement analysis. Interobserver agreement was assessed using Bland–Altman plots and with ICCs of a two-way random model with absolute agreement for single measurements (type ICC (2,1)). The paired t-test was used to assess whether there was a systematic difference between both observers.

### Ethics

The protocol was reviewed and approved by our institutional Review Board (METC: #2017-0122). Volunteers were informed about the study in word and writing. Written informed consent was obtained from all participants. The study was performed in accordance with the Declaration of Helsinki.

## Results

A total of 49 volunteers participated in this prospective study. Table 1 shows volunteer characteristics. Forty-six of the volunteers completed all 6 measurements (three for each observer). Three volunteers had one measurement less due to technical problems with the GlycoCheck.

**Table 1:** Characteristics of the 49 healthy volunteers.

Characteristic	Value
Age (years)	24 ± 4
Female; N (%)	23 (46.9%)
Height (cm)	177 ± 9
Weight (kg)	71 ± 12
BMI (kg/m^2^)	22.9 ± 3.2

Data are expressed as mean ± standard deviation.

### Intraobserver agreement

For single measurements the ICC was < 0.4 (poor) for PBR, red blood cell filling percentage and microvascular vessel density (Table 2). When three consecutive measurements were averaged the ICCs improved to ≥ 0.6 (indicating good reproducibility) for all outcomes in both observers.

**Table 2:** ICCs of experienced operators.

	Experienced observer 1 N = 48		Experienced observer 2 N = 47	
	ICC single measurement	ICC average of 3 measurements	ICC single measurement	ICC average of 3 measurements
PBR	0.35 (0.17–0.53)	0.62 (0.38–0.77)	0.38 (0.20–0.56)	0.65 (0.43–0.79)
Red blood cell filling percentage	0.37 (0.19–0.55)	0.64 (0.41–0.78)	0.36 (0.18–0.54)	0.62 (0.40–0.78)
Density	0.35 (0.18–0.53)	0.62 (0.40–0.77)	0.38 (0.20–0.56)	0.67 (0.43–0.79)

Data are expressed as intraclass correlation coefficients (95% confidence interval).

### Interobserver agreement

**Figure 1:**
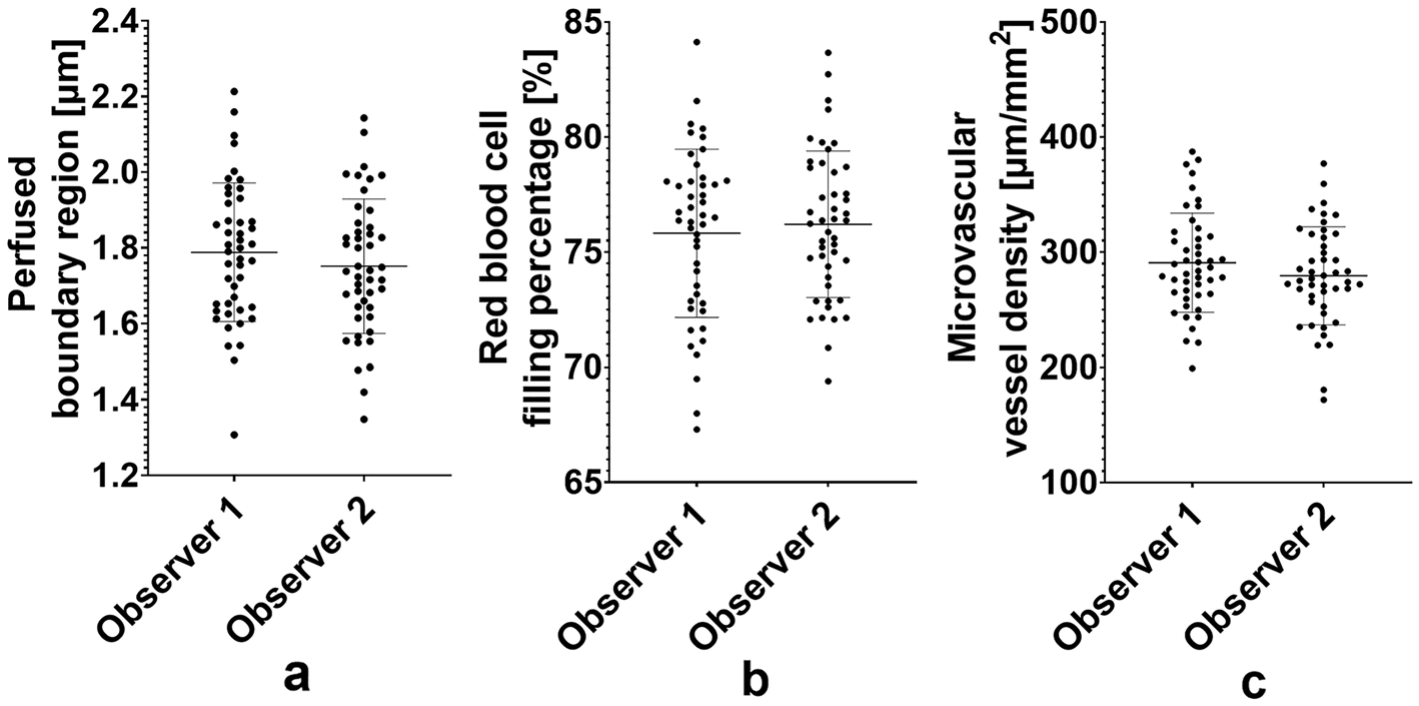
Bee swarm plots of perfused boundary region (**a**), red blood cell filling percentage (**b**) and microvascular vessel density (**c**) of the 46 volunteers measured by both observers. Lines indicate means and standard deviations. None of the variables are statistically significantly different when comparing the two observers (paired *t* test).

No systematic differences were seen between observer 1 and 2 (Fig. 1a–c) for PBR (1.79 ± 0.18 μm vs. 1.75 ± 0.18 μm; p = 0.16), red blood cell filling percentage (75.8 ± 3.7 vs. 76.2 ± 3.2; p = 0.40) and perfused vessel density (291 ± 43 µm/mm^2^ vs. 279 ± 43 µm/mm^2^; p = 0.16).

**Figure 2:**
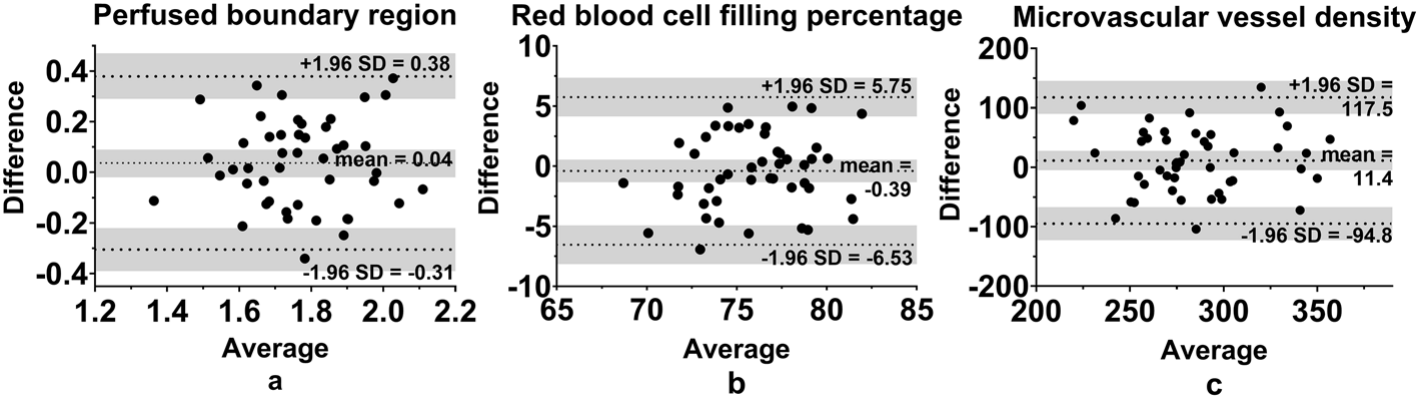
Bland Altman plots of the perfused boundary region (**a**), the red blood cell filling percentage (**b**) and the microvascular vessel density (**c**) of experienced observers 1 and 2. The dotted lines depict the limits of agreement and the mean as indicated (the grey areas indicate their 95% CI).

The ICC of the two observers for PBR and red blood cell filling percentage was 0.53 (0.29–0.71) and 0.58 (0.36–0.75) respectively, indicating fair agreement. The ICC for the perfused vessel density was 0.20 (− 0.09–0.46), indicating poor agreement. The Bland–Altman plots show limited bias, no proportional error and no inconsistent variability for PBR, red blood cell filling percentage and vessel density (Fig. 2a–c).

## Discussion

Microcirculation measurements are increasingly performed and microcirculatory derangements are negatively associated with outcome in various disease states such as sepsis and shock^17–19^. The GlycoCheck system offers a non-invasive, relatively fast, bed-side tool to assess the microcirculation and specifically the sublingual glycocalyx. Knowing the reliability of the measurements and determining optimal measuring protocols is crucial not only for future adaptation of microcirculation measurements with GlycoCheck in clinical practice, but also for defining research protocols and for comparability between individual studies. The present study was designed to assess intra- and interobserver variability of the GlycoCheck in healthy volunteers. The results showed poor intraobserver reproducibility for single measurements of PBR, red blood cell filling percentage and microvascular vessel density. Intraobserver reproducibility was improved to acceptable levels when averaging three consecutive measurements. Interobserver variability of the average of three consecutive measurements was fair for PBR and red blood cell filling percentage and poor for microvascular vessel density. No systematic differences were observed between the two observers for any of the outcome measures.

Intraobserver variability of the GlycoCheck system has been evaluated in other populations. In 30 patients admitted to the emergency room and intensive care unit, Rovas et al. found excellent intraobserver reproducibility of both the PBR and red blood cell filling percentage (ICCs of 0.77 (0.52–0.89) and 0.88 (0.74–0.94), respectively)^20^. Previously, Bol et al. reported ICCs for single measurements in 50 ICU patients of 0.57 (0.41–0.71), 0.76 (0.64–0.84) and 0.59 (0.44–0.72) for PBR, red blood cell filling percentage and microvascular vessel density, respectively, indicating fair intraobserver reproducibility for PBR and microvascular vessel density and good reproducibility for red blood cell filling percentage. Averaging three consecutive measurements resulted in excellent intraobserver reproducibility for all three outcome measures^11^. These two studies both report higher ICCs than we report in our current study. A possible explanation for this observed difference is the fact that the population of our current study consists of young, healthy volunteers whereas the above mentioned studies consisted of (severely) ill patients meaning the measurements were performed in a different measurement range. Additionally it could be hypothesized that sedated and intubated patients have less motion artifacts and better tolerate the measurements leading to less variability in the measurements. However, an analysis of factors such as the RASS score (indicating sedation level in critical ill patients) and breathing frequencies, did not show a correlation with the coefficient of variation in one of the above-mentioned studies in severely ill patients^11^.

In a study of 50 participants selected from the urban multiethnic HELIUS study cohort, Valerio et al. showed poor intraobserver reproducibility for both PBR and red blood cell filling percentage (ICCs of 0.33 (0.08–0.56) and 0.51 (0.27–0.69) respectively)^13^. In 31 smokers, Eickhoff et al. show poor ICCs for PBR and red blood cell filling percentage for single measurements of 0.30 (0.20–0.40) and 0.45 (0.34–0.56), respectively^12^. They found increasing reproducibility with increasing number of measurements: ICCs of 0.5 (0.38–0.62) and 0.45 (0.34–0.56) for the average of three measurements up to ICCs of 0.65 (0.54–0.76) and 0.71 (0.61–0.82) for the average of 5 measurements for PBR and red blood cell filling percentage, respectively. Both studies hence further emphasize the need to average several consecutive measurements to achieve sufficient reliability. The average of three measurements thus offers good reliability in healthy, mildly compromised and severely sick patients. Though averaging over even more consecutive measurements improves the reliability even further as shown by Eickhoff^12^, measurement time increases with increasing number of measurements. For wake patients this could lead to discomfort and for severely ill patients one can debate on how long the microcirculation can be considered stable.

Two experienced measurers do not yield a systematic difference and the ICCs indicate fair interobserver agreement for the PBR and the red blood cell filling percentage in our results which is in line with results seen in other populations^11,12,20^. When considering the limits of agreement of the PBR in the Bland–Altman plots (2 SD = 0.35 μm), however, the absolute difference between measurements performed by different observers is rather large compared to clinically relevant differences of 0.2 μm^18,21,22^. This suggests that the GlycoCheck system in its current form will not be able to detect gradual recovery or impairment of the glycocalyx in individual patients if performed by different operators—even when averaging three consecutive measurements—limiting its practical utility. As studies performed in other centers report similar or higher limits of agreement between two observers for the PBR, we expect these results to hold for any set of observers^12,20^. To our knowledge, however, there is no study that investigated the impact of training on the results.

The ICC of the perfused vessel density indicated poor interobserver agreement. A possible explanation hereof is that the observers do not get feedback which vessels are of sufficient quality to be detected and included in the automated analysis. If some vessels in a movie are detected and included in the analysis whereas other vessels in the same image are not, this can lead to an underestimation of the vessel density. The precise location of a measurement and the assessment of factors such as the absence of excessive saliva are subjective. This can lead to a higher variation between observers for microvascular density than for PBR and red blood cell filling percentage as these latter two factors do not rely on how many vessels are detected in a movie.

This study was performed with an SDF video-microscope. The successor of SDF, IDF, has been shown to visualize more vessels and to have a higher image quality^23^. Whether GlycoCheck software can process images recorded with IDF and whether this results in lower variability of the measurements is unknown. Additionally this research was performed with the original GlycoCheck software. Recently, a new version of the GlycoCheck software is described^24^. Even though the calculation method for PBR remains the same, a minimum of 6000 segments is needed to complete a single measurement versus 3000 segments in the original software version—this is similar to taking 2 measurements with the original software and averaging the results. Possibly, this decreases the variability of the measurements, however, comparative data are lacking.

## Conclusion

In conclusion, we showed that microcirculation measurements with the GlycoCheck software can be performed in healthy volunteers with acceptable reliability and reproducibility on a population level when averaging three consecutive measurements. Preferably, repeated measurements are performed by the same observer. The magnitude of variation in the measurements, however, suggests that gradual, clinically relevant changes within individual subjects may remain unnoticed with the GlycoCheck software in its current form.

## Data Availability

The datasets generated during and/or analysed during the current study are available from the corresponding author on reasonable request.
